# The impact of cognitive performance on quality of life in individuals with Parkinson's disease

**DOI:** 10.1590/s1980-5764-2016dn1004008

**Published:** 2016

**Authors:** Maira Rozenfeld Olchik, Annelise Ayres, Marcieli Ghisi, Artur Francisco Schumacher Schuh, Carlos Roberto Mello Rieder

**Affiliations:** 1 PhD. Department of Surgery and Orthopedics, Universidade Federal do Rio Grande do Sul, Porto Alegre, Rio Grande do Sul, RS, Brazil.; 2 MD. Postgraduate Program in Health Sciences, Universidade Federal de Ciências da Saúde de Porto Alegre, RS, Brazil.; 3 MD. Speech Therapy Course, Universidade Federal do Rio Grande do Sul, Porto Alegre, RS, Brazil.; 4 PhD. Graduate Program in Medical Sciences, Universidade Federal do Rio Grande do Sul, Porto Alegre, RS, Brazil.; 5 PhD. Movement Disorders Unit, Hospital de Clínicas de Porto Alegre, Porto Alegre, RS, Brazil.

**Keywords:** cognition, Parkinson's disease, evaluation

## Abstract

**Background:**

Evidence points to the occurrence of cognitive impairment in all stages of PD, constituting a frequent and debilitating symptom, due to high impact on quality of life and mortality of patients.

**Objective:**

To correlate cognitive performance with quality of life in PD.

**Methods:**

The sample was drawn from a Movement Disorders Clinic of a reference hospital in Porto Alegre. Inclusion criteria were: PD diagnosis, according to the United Kingdom Parkinson's Disease Society Brain Bank criteria for idiopathic PD (Hughes et al. 1992) and patient consent to participate. Patients with other neurological pathologies and those submitted to deep brain stimulation were excluded. The evaluation consisted of a cognitive testing battery (composed of eight tests for assessing cognitive performance), and a questionnaire on quality of life (PDQ-39) and depression (BDI).

**Results:**

The sample comprised 85 individuals with PD, with a mean age of 62.9 years (±10.7), mean disease duration of 10.4 years (±5.7), and mean educational level of four years (±4.3). There was a significant relationship between total score on the PDQ and all cognitive tests, showing that poor cognitive performance was correlated with poor quality of life. Moreover, a significant correlation was observed between cognitive tests and depression, H&Y, education level, and age.

**Conclusion:**

It may be concluded that the individuals with PD in this sample showed a correlation between poorer quality of life and worse cognitive performance. Poor performance was also correlated with more advanced stage, older age, low level of education and depression.

## Abstract (pt)

**Embasamento:**

As evidências apontam a ocorrência de comprometimento cognitivo em todas as fases da doença de Parkinson (DP), sendo este um sintoma não motor frequente e incapacitante, devido ao alto impacto na qualidade de vida e mortalidade dos pacientes.

**Objetivo:**

correlacionar a performance cognitiva com qualidade de vida na DP.

**Métodos:**

A amostra foi oriunda de um Ambulatório de Distúrbios do Movimento de um hospital referência de Porto Alegre. Os critérios de inclusão utilizados foram ter diagnóstico de DP e consentir em participar do estudo. Exclui-se pacientes com outras patologias neurológicas ou pacientes com estimulação cerebral profunda. A avaliação foi composta por uma anamnese, bateria de testes cognitivos (composta por oito testes), questionários sobre qualidade de vida (PDQ-39) e depressão (BDI).

**Resultados:**

A amostra foi composta por 85 indivíduos com DP, com média de idade de 62,9 anos (±10,7), média do tempo de doença de 10,4 anos (±5,7) e média de escolaridade de 7,4 anos (±4,3). Verificou-se relação significativa entre escore total do PDQ com todos os testes cognitivos, demonstrando que pior performance cognitiva está relacionada com pior qualidade de vida. Além disso, foi observado correlação significativa entre os testes cognitivos com depressão, H&Y, escolaridade e idade.

**Conclusão:**

Pode-se concluir que na presente amostra indivíduos com DP apresentaram correlação entre pior qualidade de vida com pior desempenho cognitivo. Isto também foi observado com estágio avançado da doença, idade avançada, baixa escolaridade e depressão.

## INTRODUCTION

Characterized as a neurodegenerative disease with degeneration of dopamine neurons, particularly in the substantia nigra of the midbrain, Parkinson's disease (PD) is the second-most-prevalent neurodegenerative disease worldwide.^1,2^ In Brazil, the prevalence of PD is 3.3% in the elderly.

It is estimated that about 25%-38.2% of individuals in the early stages of the disease present cognitive impairment.^3,4^ The literature has clearly established the occurrence of cognitive impairment at all stages of PD, representing a common and debilitating symptom due to its high impact on quality of life and mortality among patients.^3-7^

Although there is a heterogeneous clinical presentation of cognitive decline in PD, in most cases changes in executive function, attention, visuospatial function, working memory, episodic memory, and psychomotor speed are observed, suggesting alteration in the frontal lobe or frontostriatal circuits.^3-7^

Risk factors for cognitive dysfunction in PD include advanced age, low education, worsening of motor symptoms, rigidity, postural instability, excessive daytime sleepiness (behavioral disorder of REM sleep), visual hallucinations and cerebral white matter disease.^3,6^

Given the high prevalence of cognitive decline, its severity and the influence of motor symptoms in PD, it is important to determine whether there is a characteristic profile of cognitive changes for the different stages of PD. Thus, the aim of this study was to correlate cognitive performance with quality of life in Parkinson's disease.

## METHODS

A cross-sectional, observational, descriptive study of individuals diagnosed with Parkinson's disease was conducted. The study was approved by the Research Ethics Committee of a reference hospital in Porto Alegre, under number 120399.

**Sample.** The sample was derived from a Movement Disorders Clinic of a reference hospital in Porto Alegre. Inclusion criteria were: PD diagnosis, according to the United Kingdom Parkinson's Disease Society Brain Bank criteria for idiopathic PD,^8^ and patient consent to participate. Patients with other neurological pathologies or previous treatment with deep brain stimulation surgery were excluded.

**Procedures.** The evaluation was conducted in a single session, consisting of anamnesis, cognitive testing battery, and application of the Modified Hoehn and Yahr Scale (H&Y).^9^ The cognitive battery was drawn from the list of recommendations of the Movement Disorders Society (MDS), consisting of:

*Mini-Mental State Examination (MMSE):* score ranges from 0 to 30 points, with higher scores indicating better cognitive performance. Validated for Brazilian Portuguese, with normative reference values of ≥28 points for educational level >8 years; 26 for 5-8 years' education; 25 for 1-4 years; and 20 for illiterate individuals.^10^

*Categorical verbal fluency (categorical FAS):* evaluates the ability to search for and retrieve data established in long-term memory within a particular category, requiring organizational skills, self-regulation and working memory. Validated for Brazilian Portuguese, with the following normative values: cut-off of 9 animals for individuals with educational level of ≤8 years and cut-off point of 13 animals for an educational level >9 years.^11^

*Verbal fluency with phonological restriction (FAS):* this test assesses executive function, language, and semantic memory. It is validated for Brazilian Portuguese.^12^

*Rey Auditory Verbal Learning Test (RAVLT):* this comprises assessment of immediate memory, besides short and long-term retention. Validated for Brazilian Portuguese.^13^

*Montreal Cognitive Assessment (MoCA):* this test investigates the individual's skills in five areas: visuospatial/executive, naming, memory, attention, abstraction, and guidance. The total score is the sum of all items with a maximum of 30 (best performance). It is validated for Brazilian Portuguese.^14^

*Frontal Assessment Battery (FAB):* This battery assesses executive functions, such as conceptualization, mental flexibility, programming, sensitivity to interference, inhibitory control and environmental autonomy. The total score is the sum of all items, ranging from 0 (worst performance) to 18 (best performance). It is validated for Brazilian Portuguese.^15^

*Scales for Outcomes in Parkinson's disease - Cognition (Scopa-cog):* this instrument has been indicated as appropriate for evaluation of executive functions in individuals with PD. The battery consists of tasks that seek to evaluate the following cognitive functions: memory and learning, attention, executive functions, visuospatial functions, memory. The score ranges from 0 (worst performance) to 43 (best performance) points. It is validated for Brazilian Portuguese.^16^

*Trail Making Test (TMT):* version A allows evaluation of processing speed and visual attention. Version B is used to measure an individual's ability to manage competing sources of data, revealing flexibility and planning, and also used as a measure of working memory.^17^

*Parkinson's Disease Questionnaire-39 (PDQ-39):* the appropriate instrument for assessing the quality of life of individuals with PD, translated for use in Brazil.

*Beck Depression Inventory (BDI):* this is a scale for measuring depression composed of 21 questions with scores ranging from 0 to 3 points, totaling 63 points. On the BDI, depression is defined as scores <15, mild depression 15-20 points, moderate-to-severe depression 20-30 points, and severe depression 30-63 points.^18^

*Modified Hoehn and Yahr Scale (H & Y - Degree of Disability Scale):^9^* it is an instrument that allows the classification of disability of individuals with PD. It comprises seven stages of classification, assessing severity based on global measures of signs and symptoms.

**Statistical analysis.** A descriptive analysis of all variables was conducted, expressed as mean and standard deviation (SD). The Shapiro-Wilk normality test was applied to determine the homogeneity of variance. Spearman's correlation test was used for the variables MMSE, RAVLT, Trails A, written trails A and education. Pearson's correlation test was employed for symmetrical variables (age, disease duration, FAS, FAB, Scopa-cog). A 5% error was stipulated for all tests. The statistical program used was the Statistical Package for Social Sciences (SPSS) version 20.0.

## RESULTS

The sample consisted of 85 individuals with PD. The mean age of the subjects was 62.9 years (±10.7), mean disease duration was 10.4 years (±5.7) and mean education was 7.4 years (±4.3).

The significant correlation between quality of life and depression with cognitive performance is shown in Table 1.

**Table 1:** Correlation of quality of life and depression with cognitive tests.

Variables		PDQ-39 39.9 (±17.8)			BDI ^b^ 13.1 (±8.8)	
		p	r		p	r
MMSE*	24.50 (±4.0)	0.004	-0.324^b^		0.002	-0.326
FAS categorical*	13.45 (±4.9)	0.011	-0.284^a^		0.065	-0.203
FAS*	24.64 (±12.2)	0.007	-0.299^a^		0.004	-0.307
MOCA*	19.35 (±5.4)	<0.001	-0.406^a^		0.010	-0.279
RAVLT a (a1-a5)*	25.07 (±9.7)	0.008	-0.296^b^		0.006	-0.299
RAVLT immediate (a6)*	4.13 (±2.9)	0.074	-0.202^b^		0.012	-0.272
RAVLT recent (a7)*	3.07 (±3.1)	0.009	0.292^b^		0.004	-0.308
FAB*	11.80 (±3.7)	0.019	-0.264^b^		0.019	-0.255
Trail A (seconds)*	11.06 (±6.3)	0.001	0.378^b^		0.088	0.190
Trail B (seconds)*	59.20 (±37.6)	0.028	0.339^b^		0.187	0.203
Scopa-cog*	14.00 (±4.9)	0.002	-0.345^a^		0.036	-0.233

a Pearson’s correlation;

b Spearman’s correlation;

* Results expressed as mean and standard deviation; r: correlation coefficient; p: p value; H & Y: Hoehn and Yahr Scale; MMSE: Mini-Mental State Examination; FAS: Verbal Fluency; Montreal Cognitive Assessment: MOCA; FAB: Frontal Assessment Battery; RAVLT: Rey Auditory Verbal Learning Test; Scopa-cog: Scales for Outcomes in Parkinson’s disease-Cognition.

The correlation between the variables age, level of education and disease duration with the results of cognitive tests is shown in Table 2. The correlations between the H&Y scale stages and cognitive tests are given in Table 3.

**Table 2:** Correlation of age, education and disease duration with cognitive tests.

Variables	Mean (SD)	Age**			Educational level*			Disease duration**	
		p	r		p	r		p	r
MMSE*	24.50 (±4.0)	0.098	-0.182		<0.001	0.438		0.381	-0.115
FAS categorical**	13.45 (±4.9)	0.018	-0.258		<0.001	0.510		0.647	-0.060
FAS**	24.64 (±12.2)	0.004	-0.308		<0.001	0.552		0.260	-0.148
MOCA**	19.35 (±5.4)	<0.001	-0.385		<0.001	0.535		0.107	-0.210
RAVLT a (a1-a5)**	25.07 (±9.7)	<0.001	-0.397		0.002	0.335		0.910	0.015
RAVLT immediate (a6)*	4.13 (±2.9)	<0.001	-0.389		0.006	0.300		0.787	0.036
RAVLT recent (a7)*	3.07 (±3.1)	<0.001	-0.434		0.021	0.253		0.899	-0.017
FAB**	11.80 (±3.7)	0.001	-0.368		<0.001	0.532		0.248	-0.152
Trail A (seconds)*	11.06 (±6.3)	0.001	0.371		0.001	-0.437		0.789	0.036
Trail B (seconds)	59.20 (±37.6)	-	-		-	-		-	-
Scopa-cog**	14.00 (±4.9)	0.002	-0.331		<0.001	0.475		0.706	-0.050

* Spearman’s correlation;

** Pearson’s correlation; r: correlation coefficient; p: p-value; H & Y: Hoehn and Yahr Scale; MMSE: Mini Mental State Examination; FAS: Verbal Fluency; Montreal Cognitive Assessment: MOCA; FAB: Frontal Assessment Battery; RAVLT: Rey Auditory Verbal Learning Test; Scopa-cog: Scales for Outcomes in Parkinson’s disease-Cognition.

**Table 3:** Correlation between PD stage and cognitive assessment.

Cognitive Tests	H&Y	Min	Max	Percentiles			p
				25	50	75	
MMSE	2	10	30	23.25	26	27	0.09
	3	16	30	21	23	26.5	
	4	10	27	14	20	26.5	
FAS categorical	2	4	31	11.5	14.5	19	0.077
	3	3	22	10	11	17	
	4	4	18	6	10	14	
FAS	2	4	54	20	26	35.5	0.09
	3	3	49	13.5	22	35	
	4	2	39	3	16	28.5	
MOCA	2	7	26	18.5	22a	23.75	0.04
	3	9	30	14	17ab	22	
	4	3	21	7.5	14b	20	
RAVLT a (a1-a5)	2	9	47	18	25	28.75	0.602
	3	7	48	14	23	28.5	
	4	12	26	12.5	25	26	
RAVLT immediate (a6)	2	0	9	2.25	3	5	0.357
	3	0	11	1.5	3	6	
	4	0	5	0.5	1	4	
RAVLT recent (a7)	2	0	9	0.25	3	4	0.509
	3	0	10	0	1	4.5	
	4	0	5	0	0	4	
FAB	2	2	17	9.25	13a	15	0.025
	3	5	18	8	11ab	13	
	4	3	12	3	7b	12	
Trail A	2	4	46	7	8	12.75	0.063
	3	6	888	8	11	15	
	4	12	15	12	13	15	
Trail B	2	19	185	23	38	79	0.25
	3	24	142	33	48	66.5	
	4	63	97	63	79	.	
SCOPA	2	5	25	13	16a	18	0.01
	3	3	22	9	12b	14.5	
	4	4	16	4.5	11ab	15.5	

Kruskal-Wallis test; min: minimum; max: maximum; p: p-value; Different letters represent statistically different distribution. H & Y: Hoehn and Yahr Scale; MMSE: Mini-Mental State Examination; FAS: Verbal Fluency; Montreal Cognitive Assessment: MOCA; FAB: Frontal Assessment Battery; RAVLT: Rey Auditory Verbal Learning Test; Scopa-cog: Scales for Outcomes in Parkinson’s disease- Cognition.

The Trail Making Test B version was excluded from the analysis because 67.0% of individuals failed in less than five minutes (300 seconds), which is the maximum time to record the test score. Thus, only a small number of patients remained for performing statistical correlations.

## DISCUSSION

This study sought to apply a broad cognitive battery composed of eight tests, given the literature affirms the need to conduct a battery of comprehensive neuropsychological tests for accurate diagnosis of cognitive changes in PD.^19^ This is justified by the heterogeneity of clinical presentation of cognitive decline in PD, which can cause changes in several cognitive functions.^3-7^

According to the literature,^3,6,19-21^ cognitive changes are a significant non-motor symptom in individuals with PD and significantly contribute to poor quality of life. Thus, it was important to study the impact of symptoms in our population as the cognitive performance data showed different impacts on quality of life. Data indicates that 15%-25% of newly diagnosed PD patients have mild cognitive impairment (MCI), and approximately 50% of those with PD develop dementia within the first ten years of diagnosis, rising to over 80% 20 years after diagnosis.

According to the results for the sample, a significant inverse correlation was found between quality of life (PDQ-39) and cognitive tests (MMSE, Fascat, FAS, MOCA, RAVLT, FAB and Scopa-cog) and a direct correlation between QOL and the trails test. Thus, individuals with better quality of life had better cognitive performance. The same was observed for depression, which showed a significant inverse correlation between the BDI test, and the MMSE cognitive tests, FAS, MOCA, RAVLT, FAB, and Scopa-cog, demonstrating that individuals with major depression had poorer performance on the cognitive assessments.

These findings corroborate evidence in the literature^22-26^ showing that worse quality of life and depression are associated with worse cognitive performance or presence of dementia in individuals with PD. In the studies,^22-25^ there was a significant correlation between quality of life measured by PDQ-39 or PDQ- 8 and better performance on cognitive assessments.

Regarding depression, Klepac et al. (2008)^22^ observed a correlation between lower scores on the BDI with better scores on cognition and quality of life. Ng et al. (2015)^25^ found lower scores on cognitive tests in individuals with PD and depression when compared to patients without depression and to healthy controls. Furthermore, Wang et al. (2014) found that depression is a predictive risk factor for cognitive impairment in PD with an OR=1.98 and p=0.03.

Furthermore, the results revealed a positive correlation between the MMSE, FAS, FAS categorical, MOCA, RAVLT, FAB and Scopa-cog and education, and a negative correlation of the Trail Making Test with education. Thus, the higher the educational level of individuals, the better the performance on cognitive tests. Regarding age, there was a negative correlation with the FAS, FAS categorical, MOCA, RAVLT, FAB and Scopa-cog and a positive correlation with the Trail Test. Thus, the higher the age of the individual worse their performance on cognitive tests.

According to the literature, age is the biggest risk factor for developing dementia in PD.^19,20^ The key element is the current age of the patients as opposed to age at disease onset. Also, low educational level is also reported as a risk factor for dementia in this population.^19^ This justifies the correlation of older age and low education with worst performance on cognitive tests found in this sample. These data corroborate the Kandiah et al. (2014)^3^ study which found that patients with advanced age and lower education had poorer performance on cognitive tests.

The factors advanced age and lower educational level were associated with worse cognitive performance in this sample, confirming the data found in the Hindle et al. (2015)^20^ longitudinal cohort study in individuals with PD. In the cited study, higher levels of education, socioeconomic status, and recent social engagement were associated with better overall cognition in the first evaluation. After four years, normal cognition at baseline and higher levels of education were associated with better overall cognition over time. Older age and lower social commitment levels were associated with an increased risk of dementia. Individuals who developed dementia during the four years were older, had more severe motor symptoms and used the phone less at baseline.^20^

In addition to age and educational level, other risk factors have been found for cognitive dysfunction in PD, including lower scores on performance tests, rigidity, postural instability, increased daytime sleepiness and white matter disease.^3,6,27,28^

Regarding disease stage in our sample, there was a significant correlation between the H&Y and the MOCA, FAB and Scopa-cog cognitive tests, which are batteries assessing specific functions in the frontal lobe. According to the results, individuals with more advanced disease stage had poorer performance on the cited tests. These findings corroborate the literature which found a higher percentage of individuals with mild cognitive decline and moderate stages 3, 4 and 5 on the H&Y scale when compared to individuals without cognitive decline.^3,29^

In addition, these results corroborate the study by Varalta et. al. (2015)^4^ conducted in 21 individuals with PD, which found a significant correlation between balance ability and executive functions, cognitive impairment and ability to switch attention between two tasks, functional mobility and cognitive impairment, and verbal fluency and the ability to switch attention between two tasks. However, better cognitive performance was observed on the tests conducted when compared to this study, reporting median values of 14 points on the FAB, 22 on the MOCA, and 29 points on the MMSE. These differences may be explained by the higher mean years of education in the study^4^ (10.6 years) compared to the present sample (7.4 years).

Furthermore, according to literature, cognitive decline in PD is more commonly associated with dysfunction in a single cognitive domain than multiple cognitive domains. Impairments in executive function, visuospatial function, attention, memory and psychomotor speed, suggest a frontal or frontostriatal change as the cause of these cognitive deficits.^3,5,19,21^ In our study population, cognitive dysfunction in multiple domains was observed, uncompensated by low educational impact.

Finally, we conclude that poor cognitive performance among individuals with PD was correlated with worse quality of life in this sample. This poor performance was also associated with more advanced stage, older age, lower level of education and depression.
